# Penicillin Resistance of Nonvaccine Type Pneumococcus before and after PCV13 Introduction, United States

**DOI:** 10.3201/eid2306.161331

**Published:** 2017-06

**Authors:** Cheryl P. Andam, Colin J. Worby, Ryan Gierke, Lesley McGee, Tamara Pilishvili, William P. Hanage

**Affiliations:** Harvard T.H. Chan School of Public Health, Boston, Massachusetts, USA (C.P. Andam, C.J. Worby, W.P. Hanage);; Centers for Disease Control and Prevention, Atlanta, Georgia, USA (R. Gierke, L. McGee, T. Pilishvili)

**Keywords:** Streptococcus pneumoniae, pneumococcus, penicillin, antibiotic resistance, antimicrobial resistance, geographic variation, temporal variation, serotype, nonvaccine type, 13-valent pneumococcal conjugate vaccine, PCV13, bacteria, bacterial infection, vaccines, United States

## Abstract

Introduction of 13-valent pneumococcal conjugate vaccine in the United States was not associated with a significant change in prevalence of penicillin resistance in nonvaccine type serotypes because of the variable success of highly resistant serotypes. Differences in regional serotype distribution and serotype-specific resistance contributed to geographic heterogeneity of penicillin resistance.

*Streptococcus pneumoniae* (pneumococcus) causes a range of debilitating and potentially life-threatening infections, such as pneumonia, meningitis, and septicemia. To reduce illness and death caused by pneumococcal diseases, a 7-valent pneumococcal conjugate vaccine (PCV7) was introduced in 2000 and targeted serotypes 4, 6B, 9V, 14, 18C, 19F, and 23F. However, although vaccine type serotypes declined in frequency after PCV7 introduction (*1*,*2*), an increasing frequency of nonvaccine type (NVT) serotypes in samples from carriage and invasive disease was observed in subsequent years (*2*,*3*). Known as serotype replacement, this population-level change in serotype distribution, which most often involves preexisting clones and serotypes that were already in circulation before vaccine implementation (*4*), can reduce the benefits of vaccination (*5*). To address the rise in invasive pneumococcal disease associated with NVT serotypes, a second-generation conjugate vaccine was implemented in 2010 (PCV13), targeting the 7 serotypes targeted by PCV7 plus 6 additional serotypes: 1, 3, 5, 6A, 7F, and 19A (*6*).

The prevalence of penicillin-resistant pneumococcus strains varies considerably between states (*7*,*8*). Such variation might be caused by differences in serotype distribution (such that some locations have a higher prevalence of strains that are generally more resistant) or higher-than-average levels of resistance within serotypes. Before the introduction of PCV7, regional variations in the prevalence of antibiotic resistance were considered to be caused by regional differences in antibiotic use, leading to differences in the intensity of selective pressure acting on the bacterial population (*9*). The variation in the proportion of resistant isolates within individual serotypes in the United States was thought to be a reflection of this regional difference in antibiotic use and was identified as the major factor in driving geographic variation of penicillin resistance (*7*). However, post-PCV7, this factor played a diminishing role in explaining geographic heterogeneity in penicillin resistance, with variation in serotype distribution between sites being of increasing importance (*8*). Understanding the underlying causes of the geographic heterogeneity of penicillin resistance and the role of selective pressure provides important insights on the long-term dynamics of penicillin resistance in the United States.

## The Study

To analyze NVT penicillin-nonsusceptible pneumococcus (PNSP) detected in patients with invasive pneumococcal disease, we used data from the Active Bacterial Core surveillance (ABCs) system, a population- and laboratory-based collaborative system between the Centers for Disease Control and Prevention and state health departments and academic institutions in 10 states (California, Colorado, Connecticut, Georgia, Maryland, Minnesota, New Mexico, New York, Oregon, and Tennessee). We considered PNSP non-PCV13 serotypes detected in patients in all age groups from 2009 (pre-PCV13, n = 285 patients) through 2012 (post-PCV13, 339 patients). Nonsusceptibility was based on the meningitis breakpoint (MIC >0.12 μg/mL), as recommended by the Clinical and Laboratory Standards Institute (*10*). Serotypes 15B, 15C, and 15B/C were grouped together as 15BC because of the reported reversible switching between the 2 serotypes, which makes the precise differentiation of these serotypes difficult (*11*).

**Figure 1:**
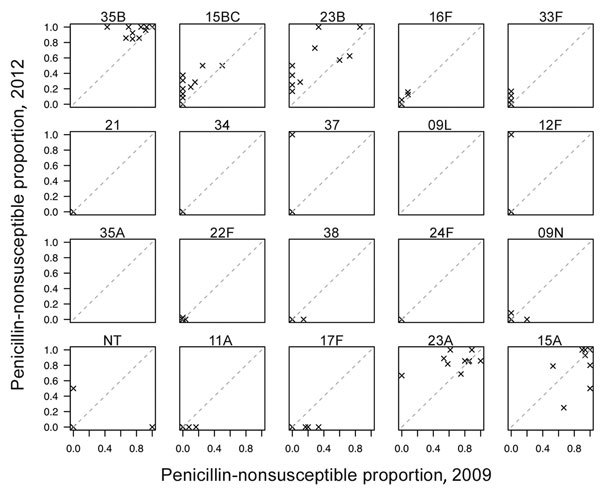
Comparison of proportion of nonvaccine type serotypes with penicillin resistance, by serotype, United States, 2009 and 2012. Based on Active Bacterial Core surveillance system data from 10 US states. The dashed diagonal line represents no change.

To determine whether geographic differences in the proportions of PNSP were consistent across serotypes, we calculated the proportions of PNSP for each of the 7 most common NVT serotypes (15A, 15BC, 16F, 23A, 23B, 33F, and 35B) across the 10 sites for 2009 and 2012. We found that serotypes with the highest proportions of PNSP in 2012 already had high resistance in 2009 (Figure 1). We calculated the Spearman correlation coefficient between the proportion of PNSP for each pair of serotypes across states in 2009 (range –0.09 to 0.66) and 2012 (range 0.30–0.79) (Technical Appendix). We found significant overall correlation in 2009 and 2012 (p<0.001 for both years), indicating that sites with high proportions of PNSP in 1 serotype typically will also have high proportions of PNSP in other serotypes. This finding suggests that differences in selection pressure account for the geographic variation in the proportions of PNSP.

**Figure 2:**
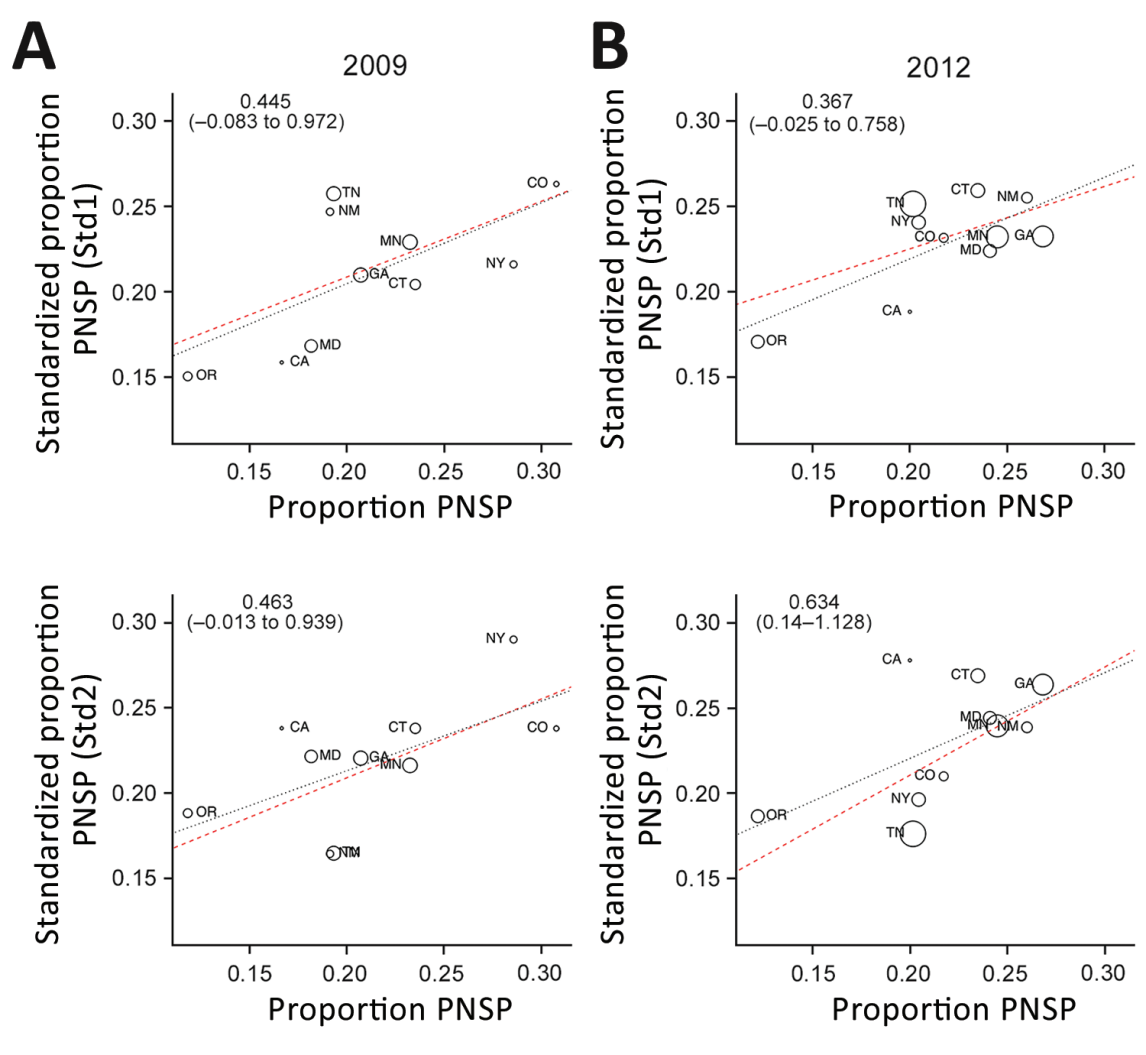
Crude versus standardized proportions of nonvaccine type serotypes with penicillin nonsusceptibility, by state, United States, 2009 and 2012, based on Active Bacterial Core surveillance system data from 10 US states. Std1 denotes standardization for geographic heterogeneity in serotype distribution. Std2 denotes standardization for serotype-specific differences in resistance. Regression slopes with 95% CIs are indicated in the upper left corner of each panel. Larger circles represent states with a greater number of penicillin-resistant samples. Dashed lines represent the inverse-variance weighted (red) and unweighted (gray) regression slopes. PNSP, penicillin-nonsusceptible pneumococcus.

We next implemented a standardized regression approach, used previously to analyze the pneumococcal-resistance patterns pre-PCV7 (*7*) and post-PCV7 (*8*) (Technical Appendix). To investigate the source of geographic variation in the proportion of PNSP, we tested the hypotheses that either geographic heterogeneity in serotype distribution (Std1), or serotype-specific differences in penicillin resistance (Std2) were responsible for the observed variation. These effects were quantified by regressing crude versus standardized prevalence of penicillin resistance (Figure 2; Technical Appendix), by which a regression slope of 1 would indicate that the factor considered had zero effect. By using 2009 data, we found that regression slopes for Std1 (0.445, 95% CI –0.083 to 0.972) and Std2 (0.463, 95% CI –0.013 to 0.939) indicate that both factors played a similar intermediate role in generating this geographic variation in penicillin resistance, with neither 95% CI containing 1. In 2012, the regression coefficient for Std2 was higher (0.634, 95% CI 0.14–1.128), whereas the coefficient for Std1 decreased (0.367, 95% CI –0.025 to 0.758). Although these changes are not statistically significant relative to 2009, they might suggest shifting contributions to the observed variation in proportions of PNSP after the introduction of the PCV13 vaccine in 2010, with geographic differences in serotype distribution having an increased role and differences in serotype-specific PNSP becoming less important.

Finally, we sought to quantify the rate of change in penicillin resistance during 2009–2012 in each state. We documented the proportion of PNSP by state for the pre- and post-PCV13 periods (Technical Appendix Table 4). No significant change in state-level resistance was observed. New Mexico, Maryland, and Georgia saw the highest increases in the proportion of PNSP during 2009–2012, whereas a slight decline was observed for Colorado, New York, and Connecticut. Although the distribution of serotypes might greatly fluctuate among geographic regions immediately after vaccine introduction, the overall proportions of NVT serotypes with penicillin resistance across the country might not vary significantly between the pre- and post-vaccine periods. Of potential importance are the small increases in the proportions of PNSP serotypes not included in either vaccine that were observed between the implementation of PCV7 and PCV13 (*12*), which might lay the foundation for changes post-PCV13.

## Conclusions

The marked variation in the proportion of penicillin resistance among states highlights the potential of local selective pressures to favor certain serotypes and resistant strains within each serotype to increase in frequency as the population returns to equilibrium (*13*). Previous studies have already shown significant regional differences in antibiotic use and vaccination coverage across the United States (*14*,*15*). Regional rates of patient adherence to treatment regimens will also influence variations in resistance. A combination of these factors, which will likely vary between and within regions, would greatly affect proportions of resistance across the country. 

In our study, we observed that the dynamics of penicillin resistance continue to shift in the wake of vaccine introduction. Our postvaccine observations were recorded shortly after the introduction of the vaccine; additional observations would be valuable to determine the stability of the postvaccine dynamics and any potential importance of the temporal changes we observed to factors contributing to variation in resistance levels. Further long-term nationwide surveillance of serotype dynamics is required to assess the multiple ecologic factors that influence antibiotic resistance in the pneumococcus in the conjugate vaccine era.

Technical Appendix Methods and additional results for a study assessing penicillin resistance of nonvaccine type pneumococcus before and after PCV13 introduction, United States.
