# Supplementary material for: Penicillin Resistance of Nonvaccine Type Pneumococcus before and after PCV13 Introduction, United States
Source: Emerg Infect Dis. 2017 Jun;23(6):1012–5. doi: 10.3201/eid2306.161331 (PMC5443431; doi:10.3201/eid2306.161331)
Supplement: Technical Appendix — Methods and additional results for a study assessing penicillin resistance of nonvaccine type pneumococcus before and after PCV13 introduction, United States. [file 16-1331-Techapp-s1.pdf]

Article DOI: https://dx.doi.org/10.3201/eid2306.161331

Penicillin Resistance of Nonvaccine Type Pneumococcus before and after PCV13 Introduction, United States

Technical Appendix

Methods

We implemented the standardization method used by McCormick and colleagues to analyze the pre-PCV7 pneumococcus dataset from the ABCs system (*1*). The same method was also used by Link-Gelles and colleagues to compare the pre-PCV7 and post-PCV7 pneumococcal populations (*2*). In both studies, standardized proportions were calculated to reflect either the differences in serotype composition or the differences in proportions of resistance against penicillin and erythromycin within individual serotypes, in their contribution to the prevalence of PNSP at each sample site.

The observed prevalence of resistance may vary by state for several reasons. Antibiotic usage across the ten states is highly non-uniform, and resistance to penicillin and other antibiotics has been shown to be significantly associated with prescription rates (*3*). If there were geographic heterogeneity in selective pressure due to varying antibiotic usage, one would expect correlation in the prevalence of penicillin resistance between serotypes (*2*). To test this, we calculated the Spearman's correlation coefficient c_{ij} between the proportion of penicillin resistance for each pair of serotypes i and j across states in both 2009 and 2012. We considered the seven most common NVT serotypes (15A, 15BC, 16F, 23A, 23B, 33F, 35B). While other NVTs were also present, none were significant contributors to the PNSP population at any study site. Taking the sum of these coefficients

$$C = \sum_{i,j} c_{ij}$$

as a test statistic, we could assess the significance of site-specific selective pressure using a permutation test to determine whether correlation was greater than would be expected at random.

We next investigated to what extent observed differences in penicillin resistance prevalence could be attributed to:

1. Spatial heterogeneity in serotype distributions (Std1),

2. Serotype-specific prevalence of penicillin resistance (Std2).

We standardized the observed prevalence of penicillin resistance in each state, adjusting for each of the two factors Std1 and Std2 (*1*,*2*). We performed an inverse-variance weighted linear regression between the crude observations and the standardized values. If the observed and standardized values are identical (linear regression coefficient 1), then the factor under consideration has no effect on the observation. Conversely, if the observed and standardized values have no relationship (linear regression coefficient 0), then the factor under consideration is completely responsible for the observations. We repeated this analysis for observations in 2009 (post-PCV13) and 2012 (post-PCV13). Finally, we calculated the rate of change for prevalence of penicillin resistance between 2009 and 2012 for each state under a logistic growth model.

Results

A total of 3,746 pneumococcal isolates of all serotypes were collected in 2009 and 2,740 in 2012, of which a total of 1,312 (35.02%) and 1,488 (54.31%) were NVTs respectively. The increase in proportion of IPD cases resulting from NVTs was significant ($p < 0.05$) in all but one state (California, $p = 0.057$), which might be attributable to the removal of VT in the population although serotype replacement may also be playing a role. The five most common NVTs collected in 2009 were 22F (n = 249; 19% of all NVTs), 33F (n = 119; 9%), 23A (n = 114; 9%), 16F (n = 111; 9%) and 15A (n = 109; 9%), while 2012 was dominated by serotypes 22F (n = 333; 22%), 09N (n = 117; 8%), 33F (n = 115; 8%), 35B (n = 114; 8%) and 15BC (n = 108; 7%). The proportions of NVTs as a total of sampled IPD cases differed significantly across states both in 2009 ($p = 0.04$) and 2012 ($p < 0.001$) (Technical Appendix Figure 1).

A total of 285 (22%) NVTs were penicillin resistant in 2009 and 339 (23%) in 2012. The NVTs exhibiting by far the highest proportions of penicillin resistance in the sample were 35B, 23A, 15A, 23B and 15BC (Technical Appendix Table 1). A significant increase (from 9% to 28%, $p = 0.01$) in the frequency of isolates carrying serotype 15BC was observed between 2009

and 2012, along with non-significant increases in 35B, 23A and 23B (Figure 1; Technical Appendix Table 1). The observed prevalence of PNSP varied considerably by serotype and between sample sites. In particular, serotypes 23A, 15A and 23B showed marked changes in the proportion of isolates that were susceptible while the great majority of serotypes show no increase (or decrease) between 2009 and 2012 (Technical Appendix Figures 2 and 3).

The introduction of serotype-specific pneumococcal vaccines can result in large fluctuations in the structure and dynamics of pneumococcal populations. Serotype replacement may partially offset the benefits of vaccination over the long term until a PCV targeting additional serotypes is implemented, as was observed with PCV7 (*4*). While no replacement disease has yet been reported following PCV13, the potential for increasing resistance in NVT is of great concern, particularly since serotype replacement has been reported in pneumococcal carriage (*5*–*7*). Previous studies of NVTs have reported that vaccines have altered the population structure of NVTs through serotype replacement (*8*–*11*). During the post-PCV7 period, there was an observed increase in PNSP among NVTs (*12*–*14*), but the underlying causes are not fully understood. In serotypes 15A, 23A, and 35B, penicillin nonsusceptibility shifted in the post-PCV7 era mainly as a consequence of shifts of clonal populations within individual serotypes that were comprised of distinct susceptible and nonsusceptible clonal complexes (*13*), but other factors might explain increased resistance in other serotypes. Understanding this is critical as it is anticipated that a complete or near complete replacement of the PCV13 serotypes in carriage is likely to occur once the population has regained equilibrium, as was observed in different parts of the United States after PCV7 implementation (*5*,*15*–*17*). While the antibiotic resistance profiles of the replacing NVTs from disease are known, as NVTs become more common over time greater exposure to antibiotics may produce selection for increased resistance. In this study, we found that while prevalence of PNSP remained largely constant following PCV13 introduction, there was much geographic heterogeneity and variation by serotype, which should be monitored closely to detect emerging threats.

We implemented the standardization method used by McCormick and colleagues to analyze the pre-PCV7 pneumococcus dataset from the ABCs system (*1*). The same method was also used by Link-Gelles to compare the pre-PCV7 and post-PCV7 pneumococcal populations (*2*). In both studies, standardized proportions were calculated to reflect either the differences in serotype composition or the differences in proportions of resistance against penicillin and

erythromycin within individual serotypes, in their contribution to the prevalence of PNSP at each sample site. In contrast to the two previous studies in which the whole sampled pneumococcus population was analyzed and all serotypes were included, we standardized the geographic differences in penicillin resistance as a proportion of the NVT population only.

Two factors play an important role in producing the geographic variation in the proportion of penicillin resistance: differences in the serotype composition and differences in the proportions of penicillin resistance within individual serotypes, which is primarily due to selection of pre-existing resistant clones within serotype. Our results suggest that both have equally contributed to the observed variation before the introduction of PCV13, but serotype distribution played a slightly greater role post-vaccine. The contribution of serotype distribution is likely influenced by changes in the distribution of VT, such that antibiotic pressure is applied only to the serotypes currently circulating at any given point in time.

Our results were in contrast to a similar study conducted on the population before PCV7 use that showed geographic variation in penicillin resistance is attributable primarily to within-serotype proportions of penicillin resistance - and hence implicated differences in antibiotic use among sites (*1*). However, our results for 2009 were similar to those reported by Link-Gelles and colleagues for pneumococcus collected in the later years of post-PCV7 (2007–09) (*2*), indicating that both factors drive the geographic variation in penicillin resistance when the population is close to equilibrium.

Our standardization results for 2012 were also in direct contrast to those reported for the years immediately following PCV7 introduction (2002–06) (*2*). Although both factors remain important during these time periods, our study shows that serotype composition played a slightly greater role, while the previous study reported a greater contribution by the differences in the proportion of penicillin resistance within each serotype. Two reasons may explain the disparity between our results and those of Link-Gelles and colleagues (*2*). First, since our focus was only on NVTs, rather than both VT and NVT isolates as used in the previous study (n = 6,929), the small sampling size in our study naturally leads to increased uncertainty. Alternatively, short-term dynamics may be different between the two groups of serotypes. We can suggest that immediately following PCV implementation, there is a rapid and stochastic shuffling of different genotypes within the local population at each site as a result of the opening up of new niches

following the elimination of VTs, and which NVTs are involved at each site will depend on which happened to be present before vaccination. For the VTs remaining in the population post-vaccine, differences in the proportion of penicillin resistance within individual serotypes remain as the primary driving factor mainly because some of these VTs, despite the declining numbers, continue to be present in relatively higher frequencies when the population has still not yet reached equilibrium. This is the case for serotype 19A, which remains an important disease-causing and highly resistant serotype, even though it has declined in frequency during the early post-PCV13 period and NVTs are now primarily detected (*11*,*18*). Hence, the geographic variation in penicillin resistance of NVTs will therefore largely depend on which NVTs were present in the population before the vaccine was implemented, even if they occurred initially at very low frequencies.

One limitation of our study is that we did not consider the effects of serotype-specific differences in the proportion of penicillin resistance by age group (*14*). This variation may also be affected by differences in antibiotic pressure due to differences in antibiotic consumption between children and adults (*19*). While our study provides a quantifiable assessment of the relative contributions of serotype distribution and serotype-specific proportions of penicillin resistance in NVT, future investigation should focus on age-related differences in resistance to specifically identify the underlying causes of such variation.

References

1. McCormick AW, Whitney CG, Farley MM, Lynfield R, Harrison LH, Bennett NM, et al. Geographic diversity and temporal trends of antimicrobial resistance in *Streptococcus pneumoniae* in the United States. Nat Med. 2003;9:424–30. PubMed http://dx.doi.org/10.1038/nm839

2. Link-Gelles R, Thomas A, Lynfield R, Petit S, Schaffner W, Harrison L, et al. Geographic and temporal trends in antimicrobial nonsusceptibility in *Streptococcus pneumoniae* in the post-vaccine era in the United States. J Infect Dis. 2013;208:1266–73. PubMed http://dx.doi.org/10.1093/infdis/jit315

3. Hicks LA, Chien Y-W, Taylor TH Jr, Haber M, Klugman KP; Active Bacterial Core Surveillance (ABCs) Team. Outpatient antibiotic prescribing and nonsusceptible *Streptococcus pneumoniae* in the United States, 1996-2003. Clin Infect Dis. 2011;53:631–9. PubMed http://dx.doi.org/10.1093/cid/cir443

4. Feikin DR, Kagucia EW, Loo JD, Link-Gelles R, Puhan MA, Cherian T, et al.; Serotype Replacement Study Group. Serotype-specific changes in invasive pneumococcal disease after pneumococcal conjugate vaccine introduction: a pooled analysis of multiple surveillance sites. PLoS Med. 2013;10:e1001517. PubMed http://dx.doi.org/10.1371/journal.pmed.1001517

5. Desai AP, Sharma D, Crispell EK, Baughman W, Thomas S, Tunali A, et al. Decline in pneumococcal nasopharyngeal carriage of vaccine serotypes after the introduction of the 13-valent pneumococcal conjugate vaccine in children in Atlanta, Georgia. Pediatr Infect Dis J. 2015;34:1168–74. PubMed http://dx.doi.org/10.1097/INF.0000000000000849

6. Moore MR, Link-Gelles R, Schaffner W, Lynfield R, Lexau C, Bennett NM, et al. Effect of use of 13-valent pneumococcal conjugate vaccine in children on invasive pneumococcal disease in children and adults in the USA: analysis of multisite, population-based surveillance. Lancet Infect Dis. 2015;15:301–9. PubMed http://dx.doi.org/10.1016/S1473-3099(14)71081-3

7. Moore MR, Link-Gelles R, Schaffner W, Lynfield R, Holtzman C, Harrison LH, et al. Effectiveness of 13-valent pneumococcal conjugate vaccine for prevention of invasive pneumococcal disease in children in the USA: a matched case-control study. Lancet Respir Med. 2016;4:399–406. PubMed http://dx.doi.org/10.1016/S2213-2600(16)00052-7

8. Richter SS, Heilmann KP, Dohrn CL, Riahi F, Diekema DJ, Doern GV. Pneumococcal serotypes before and after introduction of conjugate vaccines, United States, 1999-2011(1.). Emerg Infect Dis. 2013;19:1074–83. PubMed http://dx.doi.org/10.3201/eid1907.121830

9. Lee GM, Kleinman K, Pelton SI, Hanage W, Huang SS, Lakoma M, et al. Impact of 13-Valent pneumococcal conjugate vaccination on *Streptococcus pneumoniae* carriage in young children in Massachusetts. J Pediatric Infect Dis Soc. 2014;3:23–32. PubMed http://dx.doi.org/10.1093/jpids/pit057

10. Mendes RE, Costello AJ, Jacobs MR, Biek D, Critchley IA, Jones RN. Serotype distribution and antimicrobial susceptibility of USA *Streptococcus pneumoniae* isolates collected prior to and post introduction of 13-valent pneumococcal conjugate vaccine. Diagn Microbiol Infect Dis. 2014;80:19–25. PubMed http://dx.doi.org/10.1016/j.diagmicrobio.2014.05.020

11. Kaplan SL, Center KJ, Barson WJ, Ling-Lin P, Romero JR, Bradley JS, et al. Multicenter surveillance of *Streptococcus pneumoniae* isolates from middle ear and mastoid cultures in the 13-valent pneumococcal conjugate vaccine era. Clin Infect Dis. 2015;60:1339–45. Epub 2015 Feb 3. PubMed https://doi.org/10.1093/cid/civ067

12. Farrell DJ, Klugman KP, Pichichero M. Increased antimicrobial resistance among nonvaccine serotypes of *Streptococcus pneumoniae* in the pediatric population after the introduction of 7-valent pneumococcal vaccine in the United States. Pediatr Infect Dis J. 2007;26:123–8. PubMed http://dx.doi.org/10.1097/01.inf.0000253059.84602.c3

13. Gertz RE Jr, Li Z, Pimenta FC, Jackson D, Juni BA, Lynfield R, et al.; Active Bacterial Core Surveillance Team. Increased penicillin nonsusceptibility of nonvaccine-serotype invasive pneumococci other than serotypes 19A and 6A in post-7-valent conjugate vaccine era. J Infect Dis. 2010;201:770–5. PubMed http://dx.doi.org/10.1086/650496

14. Tomczyk S, Lynfield R, Schaffner W, Reingold A, Miller L, Petit S, et al. Prevention of antibiotic-nonsusceptible invasive pneumococcal disease with the 13-valent pneumococcal conjugate vaccine. Clin Infect Dis. 2016;62:1119–25. PubMed http://dx.doi.org/10.1093/cid/ciw067

15. Hanage WP, Finkelstein JA, Huang SS, Pelton SI, Stevenson AE, Kleinman K, et al. Evidence that pneumococcal serotype replacement in Massachusetts following conjugate vaccination is now complete. Epidemics. 2010;2:80–4. PubMed http://dx.doi.org/10.1016/j.epidem.2010.03.005

16. Halasa NB, Grijalva CG, Arbogast PG, Talbot TR, Craig AS, Griffin MR, et al. Nearly complete elimination of the 7-valent pneumococcal conjugate vaccine serotypes in Tennessee. Pediatr Infect Dis J. 2013;32:604–9. PubMed http://dx.doi.org/10.1097/INF.0b013e318287fe0d

17. Sharma D, Baughman W, Holst A, Thomas S, Jackson D, da Gloria Carvalho M, et al. Pneumococcal carriage and invasive disease in children before introduction of the 13-valent conjugate vaccine: comparison with the era before 7-valent conjugate vaccine. Pediatr Infect Dis J. 2013;32:e45–53. PubMed http://dx.doi.org/10.1097/INF.0b013e3182788fdd

18. Richter SS, Diekema DJ, Heilmann KP, Dohrn CL, Riahi F, Doern GV. Changes in pneumococcal serotypes and antimicrobial resistance after introduction of the 13-valent conjugate vaccine in the United States. Antimicrob Agents Chemother. 2014;58:6484–9. PubMed http://dx.doi.org/10.1128/AAC.03344-14

19. Lee GC, Reveles KR, Attridge RT, Lawson KA, Mansi IA, Lewis JS II, et al. Outpatient antibiotic prescribing in the United States: 2000 to 2010. BMC Med. 2014;12:96. PubMed http://dx.doi.org/10.1186/1741-7015-12-96

Technical Appendix Table 1. Changes in the proportions of penicillin resistance in the most abundant resistant serotypes between 2009 and 2012

	Proportion resistant, N resistance/N total (%)	
Serotype	2009	2012
35B	61/78 (78%)	108/114 (95%)
23A	84/114 (74%)	87/103 (85%)
15A	96/109 (88%)	69/83 (83%)
23B	21/63 (33%)	28/56 (50%)
15BC	7/76 (9%)	30/108 (28%)*
Others	15/872 (2%)	18/924 (2%)

* Significant increase (Fisher exact test).

Technical Appendix Table 2. Spearman correlation coefficient between the proportion of penicillin resistance for each pair of serotypes in 2009 (red, upper diagonal) and 2012 (blue, bottom diagonal).

Serotype	35B	15BC	23B	16F	23A	15A
35B		0.5224874	0.23571183	0.59731911	0.5732115	0.4130514
15BC	0.6889201		0.64062589	0.38313051	0.11323093	–0.2709142
23B	0.766871	0.8322754		–0.09401268	–0.09526162	–0.3830342
16F	0.4709555	0.5444811	0.301017		0.5954612	0.274986
23A	0.7919866	0.671008	0.5049354	0.4351319		0.6625419
15A	0.711235	0.4483838	0.3250522	0.4276296	0.7822086	

Technical Appendix Table 3. Crude and standardized proportions of all nonvaccine type pneumococci that were penicillin resistant in 2009 and 2012, stratified by state

	2009			2012		
State	Crude	Std1	Std2	Crude	Std1	Std2
CA	0.167	0.159	0.238	0.200	0.188	0.278
CO	0.308	0.263	0.238	0.217	0.232	0.210
CT	0.235	0.204	0.238	0.235	0.259	0.269
GA	0.207	0.210	0.221	0.268	0.233	0.234
MD	0.182	0.168	0.222	0.241	0.224	0.244
MN	0.233	0.229	0.216	0.245	0.232	0.240
NM	0.191	0.247	0.164	0.260	0.255	0.239
NY	0.286	0.216	0.290	0.205	0.240	0.196
OR	0.118	0.150	0.188	0.122	0.171	0.186
TN	0.193	0.258	0.165	0.202	0.252	0.176
Regression		0.445	0.463		0.367	0.634
95% CI		(–0.083–0.972)	(–0.013–0.939)		(–0.025–0.758)	(0.14–1.128)

Std1 refers to standardization removing the effects of geographic variation in serotype distribution, while Std2 refers to standardization removing the effects of serotype-specific differences in penicillin resistance levels within individual states. CI refers to confidence interval.

Technical Appendix Table 4. Changes in penicillin resistance among nonvaccine type pneumococcal strains over time by site

State	Proportion 2009	Proportion 2012	Logistic growth rate
NM	0.191 (7)	0.260 (2)	0.132 (–0.083, 0.354)
MD	0.182 (8)	0.241 (4)	0.119 (–0.068, 0.309)
GA	0.207 (5)	0.268 (1)	0.113 (–0.035, 0.264)
CA	0.167 (9)	0.200 (9)	0.074 (–0.351, 0.5)
MN	0.233 (4)	0.245 (3)	0.023 (–0.12, 0.167)
TN	0.193 (6)	0.202 (8)	0.017 (–0.142, 0.18)
OR	0.118 (10)	0.122 (10)	0.011 (–0.311, 0.338)
CT	0.235 (3)	0.235 (5)	–0.001 (–0.179, 0.177)
NY	0.286 (2)	0.205 (7)	–0.147 (–0.343, 0.045)
CO	0.308 (1)	0.217 (6)	–0.157 (–0.382, 0.064)

The ranks of each state in both 2009 and 2012 are indicated in parentheses, with the highest proportion of penicillin resistance having a rank of 1. Confidence intervals for the growth rates are shown in parentheses. The states are ordered starting from those with the highest growth rate to the least.

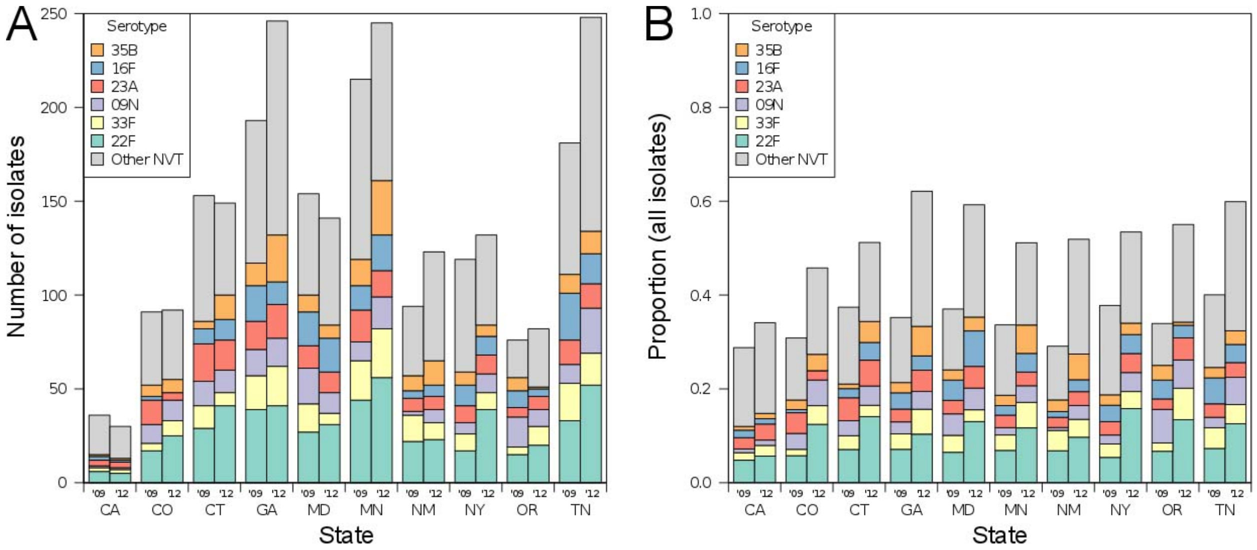

Technical Appendix Figure 1. Sampled NVTs by state and year. A) Total number of NVT isolates. B) NVTs as a proportion of the total sample. The six serotypes shown represent the most common types in the dataset.

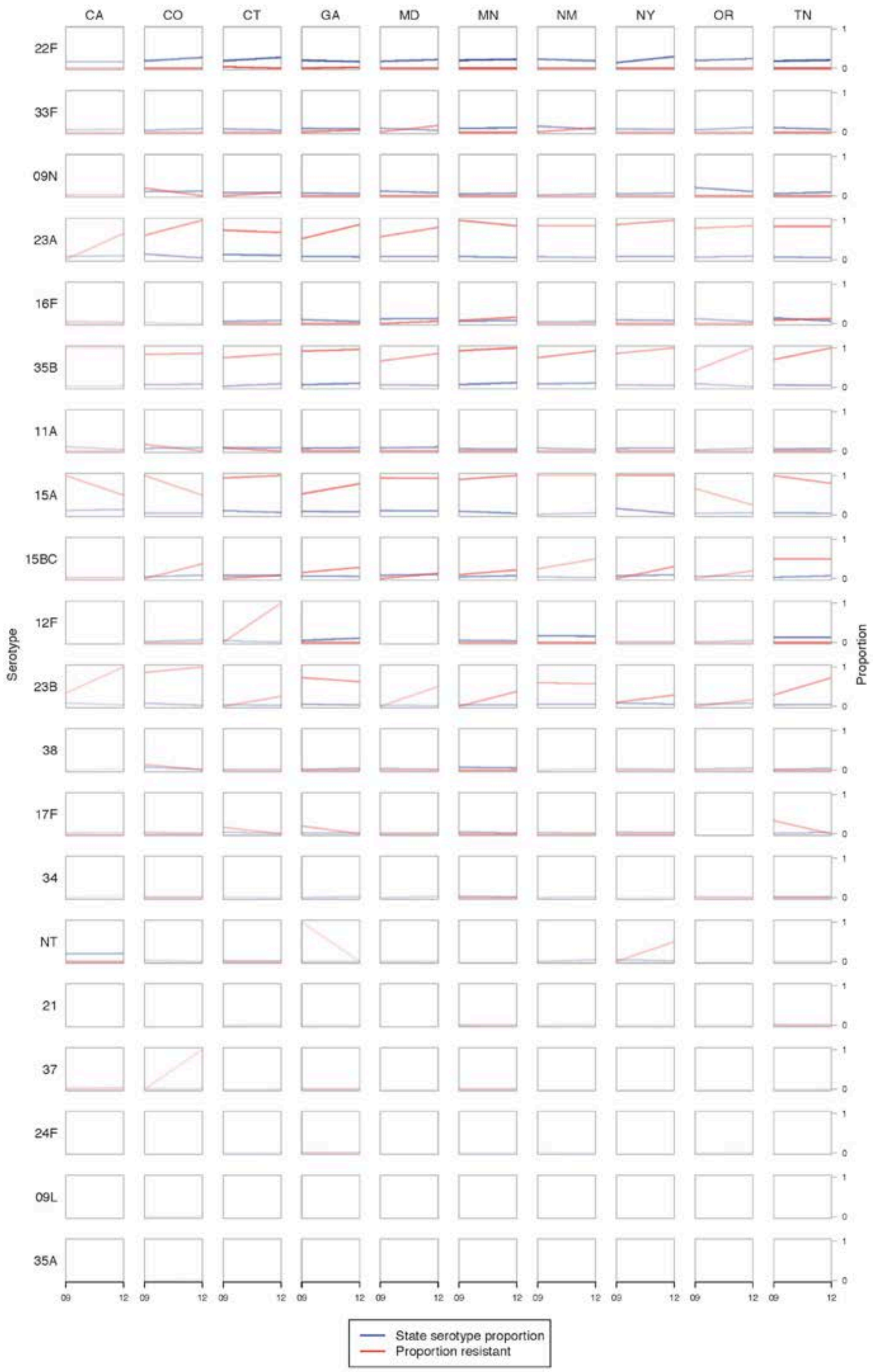

Technical Appendix Figure 2. Changes in each NVT serotype as a proportion of the total NVT population (blue) and in the proportion of penicillin resistance (red line) segregated by serotype and by state between 2009 and 2012. The weight of the line colors represent the number of isolates collected, with darker colors representing a greater number of samples.

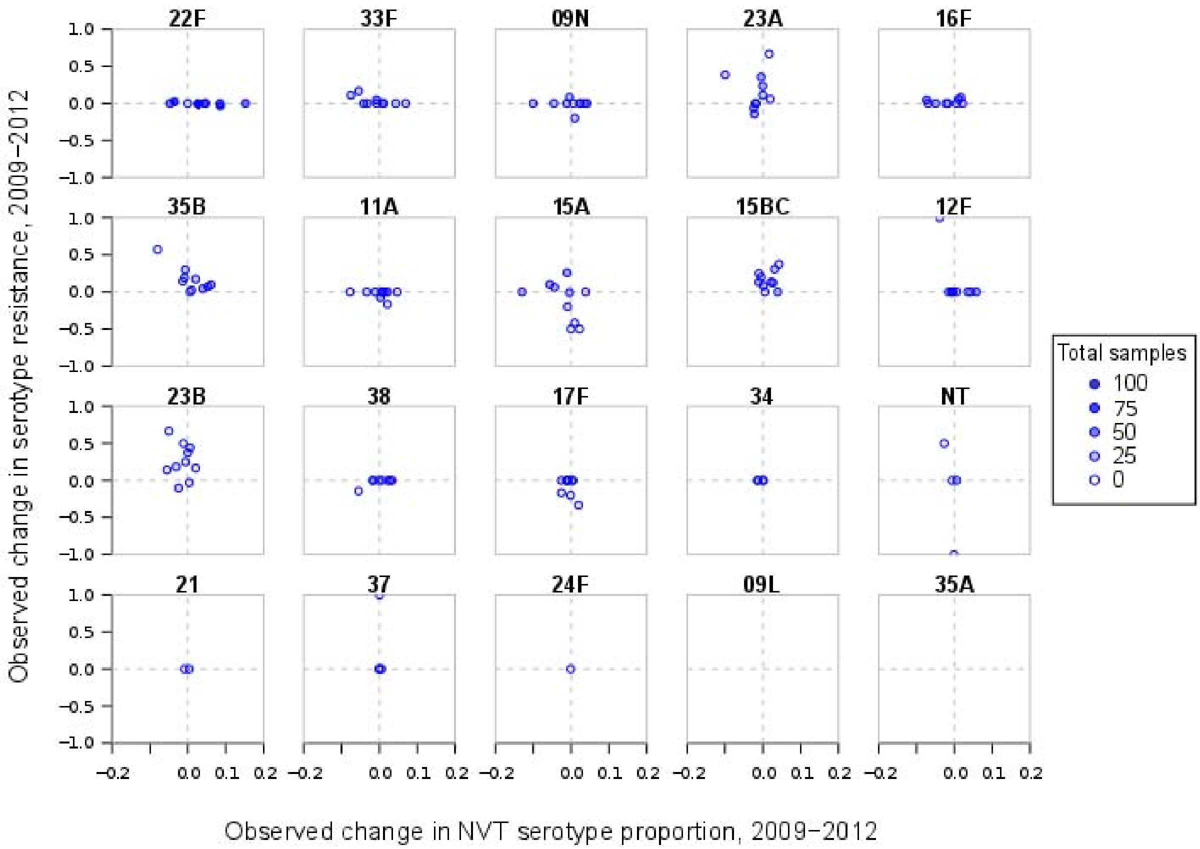

Technical Appendix Figure 3. Change in serotype proportion against change in penicillin resistance, 2009–2012. Each panel shows a single serotype, and plots for each state shows the change in proportion of NVTs represented by that serotype, and the change in prevalence of penicillin resistance between 2009 and 2012. Points are shaded to represent the combined number of samples in 2009 and 2012 for each state.
